# Cardiometabolic risk among HIV-POSITIVE Ugandan adults: prevalence, predictors and effect of long-term antiretroviral therapy

**DOI:** 10.11604/pamj.2017.27.40.9840

**Published:** 2017-05-15

**Authors:** Patrick Kazooba, Ivan Kasamba, Billy Nsubuga Mayanja, Joseph Lutaakome, Ivan Namakoola, Tino Salome, Pontiano Kaleebu, Paula Munderi

**Affiliations:** 1 MRC/UVRI Uganda Research Unit on AIDS, P.O. Box 49, Entebbe, Uganda; 2 MRC Tropical Epidemiology Group, London School of Hygiene and Tropical Medicine, Keppel Street, London WC1E 7HT, UK; 3 Department of Clinical Research, London School of Hygiene and Tropical Medicine, Keppel Street, London WC1E 7HT, UK

**Keywords:** HIV, cardiometabolic, dyslipidemia, long-term ART, hypertension, obesity

## Abstract

**Introduction:**

We investigated the prevalence, predictors of and effect of Antiretroviral Therapy (ART) regimen on cardiometabolic risk among HIV-positive Ugandan adults at enrolment into a prospective cohort to study the Complications of Long-Term ART (CoLTART).

**Methods:**

We collected data on cardiometabolic risk factors including dyslipidemia, hypertension, hyperglycemia, obesity and calculated the mean atherogenic index for Plasma (AIP) and 10 year Framingham risk score (FHS). Exposures were: ART regimen, duration on ART, demographic, socio-economic, behavioral, and life-style factors including smoking, physical activity and diet (including fruit and vegetables consumption).

**Results:**

We enrolled 1024 participants, 65% female, mean age was 44.8 years (SD 8.0) and median duration on ART was 9.4 years (IQR 6.1-9.8). The prevalence of abdominal obesity was 52.6%, BMI≥25 kg/m^2^ -26.1%, hypertension-22.6%, high AIP-31.3% and FHS above 10% was 16.6%. The prevalence of low High Density Lipoprotein (HDL) was 37.5%, high Total cholesterol (Tc)-30.2%, high Low Density Lipoprotein (LDL) -23.6%, high Triglycerides (TG)-21.2%, low physical activity-46.4% and alcohol consumption-26.4%. In multivariate linear regression analyses, increasing age was associated with higher mean Tc, HDL, LDL, FHS (P<0.001) and hyperglycemia (p<0.005). In multivariate logistic regression analyses, Protease Inhibitor (PI) containing regimens were significantly associated with higher risks of abnormal: Tc, LDL, TG, AIP, abdominal obesity, hypertension, low HDL and lower risk of a FHS >10% compared to the non PI regimen.

**Conclusion:**

ART increases cardiometabolic risk. Integration of routine assessment for cardiometabolic risk factors and preventive interventions into HIV care programs in resource-limited settings is recommended.

## Introduction

Sub Saharan Africa (SSA) has the highest burden of HIV/AIDS, with an estimated 25.8 million people living with HIV and about 1.4 million new infections in 2014 [1]. Antiretroviral therapy (ART) has improved the quality of life of people living with HIV (PLWH) and has improved life expectancy substantially [2, 3]. By June 2015, 5.8 million PLWH were receiving ART globally, of which 10.7 million were in SSA [1]. As people on ART live longer, challenges of long-term HIV infection, lifestyle changes, aging and the toxic effects of ART, are emerging. Risk factors for cardiovascular disease (CVD) among PLWH may be similar to the general population and include family history, age, male gender, hypertension, smoking, obesity, Diabetes mellitus and hyperlipidemia [4]. PLWH on ART have an estimated 10-year CVD risk greater than 20% [5]. The expected deaths attributable to CVD are projected to double to 2.4 million in 2030 relative to reports from 2000 [6]. Mortality due to CVD in SSA including Uganda is estimated to be threefold higher than in Western Europe [7]. In industrialised countries where ART has been provided for longer periods, ART agents particularly Protease Inhibitors (PI), have been associated with cardiometabolic risk factors, including impaired glucose metabolism, dyslipidemia, obesity and hypertension [8] but this has not been demonstrated in African settings. There is evidence to suggest that baseline metabolic profiles and associations between HIV, ART and cardiometabolic risk factors may differ between populations [9]. In Uganda, about 31700 deaths in 2002 were due to CVD and there is evidence of increasing CVD-related mortality from verbal autopsy data from among HIV infected people [10]. Conversely, a home-based cross-sectional study in eastern Uganda reported no effect of ART on CVD, but that study had few participants on ART, for a shorter duration and did not examine the specific effects of a PI regimen [11]. HIV prevalence in Uganda in 2014 was 7.4% and about 0.75 million (50%) of the 1.5 million PLWH were on ART of whom about 10% had HIV viral load above 1000 copies/ml [12]. The roll out of HIV viral load testing in Uganda and other African countries will increase detection of individuals with virological failure needing second line PI based ART [13]. In this study, we investigated the prevalence and predictors of, and the effect of ART on cardiometabolic risk among HIV-positive individuals who have been receiving ART for up to a decade. We specifically compared the adjusted mean differences and the risk of the various cardiometabolic risk factors between participants on a PI containing ART regimen and non PI ART regimen against the confounding effect of demographic, socio economic and behavioral factors.

## Methods

**Study design and setting**: This was a cross-sectional study that utilised data collected at enrolment into a prospective clinical cohort established in 2013 to study the Complications of Long-Term Antiretroviral therapy among HIV-Positive Ugandan adults (CoLTART). The CoLTART study aims were to describe the metabolic and renal complications, clinical and virological outcomes among HIV-positive Ugandan adults on long-term ART. However, this analysis was a sub-study specifically aimed at evaluating the cardio-metabolic complications of long-term ART. The study setting was the (i) Former Development of ART in Africa (DART) study clinic that was established in 2003 in Entebbe, Central Uganda [14], and (ii) the former Rural Clinical Cohort (RCC) that was established 1990 in south western Uganda where ART was introduced in 2004 [15].

**Study population**: The study sample selection was based on non-probability sampling of all HIV-positive adults aged 18 years and above, from two former HIV cohorts; the DART trial and RCC, who were receiving ART and consenting to undergo all study procedures. Study information was given to potential participants by a study clerk. Individuals who were too sick to undergo study procedures or unable to give consent were excluded. Participants were either taking a PI containing ART regimen or Non PI containing regimen. Non PI regimen included a standard two Nucleoside Reverse Transcriptase inhibitor (NRTI) and one Non-NRTI. Some participants from the DART trial cohort were taking a triple nucleoside therapy consisting of 3 NRTI agents. The PI containing regimen consisted of a ritonavir boosted PI mainly Lopinavir in combination with one or two NRTI.

**Measurements**: Socio-demographic, socio-economic status, medical history, behavioral and dietary exposures was obtained by self-report using a structured questionnaire. We used a modified WHO stepwise approach to surveillance questionnaire to collect cardiovascular risk data [16] (Organization). ART history data including initiation date and regimen substitutions and/or switches were obtained from the electronic databases of the two former ART cohorts. We measured the body weight using the Seca digital measuring scale and height using a portable Seca 213 Leicester stadiometer. Waist, hip and mid-upper arm circumferences were measured using a non-stretchable Seca 201 Ergonomic Circumference Measuring tape. Details of the methods for taking biophysical measurements and calibrating the equipment used have been described elsewhere [10]. Blood pressure and pulse rate were measured using the Omron M6 comfort automatic blood pressure monitor. Fasting blood glucose (FBG), Low Density Lipoprotein (LDL), High Density Lipoprotein (HDL), Total Cholesterol (Tc) and Triglycerides (TG) were measured using the Cobas Integra 400 plus (Roche Diagnostics) as described elsewhere [17]. Viral loads were quantified using the Cobas Ampliprep/Taqman V2.0 HIV-1 viral load assay [Roche Molecular Diagnostic (RMD) ,NJ,USA] with a lower limit of detection of 20 copies/ml. CD4+ cell counts were analysed using FACSCount or FACSCalibur machine [Becton Dickson, USA].

**Variables and definitions**: Cardiometabolic risk was defined as a high lifetime risk for CVD caused by specific factors including hypertension, dyslipidemia, hyperglycemia and obesity [18]. Hypertension was defined as systolic blood pressure (SBP)>140 mmHg and/or diastolic blood (DBP) pressure>90 mmHg or being on anti-hypertensive medication [19, 20]. Dyslipidemia was defined as HDL<1 mmol/L, LDL>3.4 mmol/L, Tc>5.2 mmol/L, TG>1.69 mmol/L or Tc-HDL ratio>5.1 or being on lipid lowering medicine [19, 20]. Hyperglycemia was defined as fasting blood glucose >7mmol/L or being on treatment for diabetes mellitus. Abdominal obesity was assessed using waist-hip ratio (WHR) cutoffs of 0.95 for men and 0.85 for women [21] or waist circumference (WC)>94 cm for men and>80cm for women. Abnormal BMI was defined as BMI>25 Kg/m^2^. Atherogenic index of plasma (AIP) was calculated as log10 (triglycerides/HDL). AIP of -0.3-0.1 was regarded as low risk, 0.1-0.24 medium risk and >0.24 high risk [22]. We calculated the 10 year Framingham risk score (FHS) using risk calculator graphs based on the Joint British Societies risk prediction charts [23]. We entered variables: age, gender, smoking status, diabetes status, HDL, Tc, SBP, and DBP in the online calculator tool. We chose a FHS of >10% to denote moderate to high risk of CVD. Socio demographic risk factors which included age, sex, marital status, level of formal education and occupation were assessed using subjective categories. Socioeconomic status (SES) was assessed using a household asset score (in tertiles). Behavioral and dietary risk factors including physical activity, alcohol intake, smoking, consumption of animal protein, fruit, vegetables, sugar and salt were categorised by frequency and intensity. ART regimen was categorized as PI containing or non PI containing. Baseline viral loads and CD4 counts were analyzed using subjective categories.

### Statistical methods

**Sample size considerations**: We used results from a recent survey (round 22) on metabolic abnormalities in the background population (GPC) [10] as a basis for power calculations for different proportions of participants with a metabolic abnormality among those on a PI-containing ART regimen assuming that 10% of the participants in the non-PI containing ART regimen group have this abnormality. We also assumed a within group standard deviation of: (a) 1.0 mmol/L for Tc, (b) 0.8 mmol/L for LDL, (c). 0.5 mmol/L for HDL (d) 0.8 mmol/L for TG, (e) 1.6 mmol/L for FBG (f) 19 mm Hg for SBP and (g) 12 mm Hg for DBP. We assumed having at least 200 participants on a PI-containing ART regimen and at least 800 participants on a non PI ART regimen would provide sufficient power with which a given between group mean difference will be detected as statistically significant at the 5% level.

**Statistical analysis**: Analyses were done in STATA 13 (Stata Corporation, College Station, USA). We summarized participants' characteristics at enrolment into the study and calculated the prevalence of the cardiometabolic risk factors. We compared the mean values of SBP, DBP, Tc, HDL, LDL, TG, HDL, AIP and FBG and FHS between PI-based and non-PI based ART regimens using general linear regression models, adjusted for duration on ART, age, sex, site, tobacco and alcohol consumption, physical activity, dietary variables, household asset score and CD4 cell count. Logistic regression was used to compare risks of hypertension, obesity and abnormal values for Tc, LDL, FBG, AIP, FHS, HDL and TG between the PI and non-PI based ART regimens adjusted for the same factors that were used in the linear models.

**Ethical considerations**: The CoLTART study was approved by the Research and Ethics Committee of the Uganda Virus Research Institute and by the Uganda National Council for Science and Technology. All Participants gave signed or thumb-printed written informed consent. Participants were reimbursed their transport expenses and compensated for their time during the study procedures

## Results

**Baseline characteristics of study participants**: Between July 2013 and August 2014, 1024 participants were enrolled. Majority were females (65%), most were aged 40 years and above (mean age 44.8, SD 8.0), 76% were on ART for 5 to 10 years (median 9.4 years, IQR 6.1-9.8). Participants were mainly self-employed or small scale business owners (40%), 45% had incomplete primary or no education and 42% were of low SES. The prevalence of current tobacco consumption was (7.8%), current alcohol consumption (26.4%), consumption of animal protein>3 days a week (27.8%) and low intensity physical activity (46.4%). At enrollment 23% of the participants were on PI based ART regimen, 31.5% had CD4 counts below 350 cells/ml and 17.9% had HIV viraemia above 1000 copies/ml. More men than women reported: current smoking (15.6% vs 3.7%), current alcohol consumption (38.3% vs 20.1%), consuming animal protein on > 3 days per week (33.0% vs 25.1%) and moderate to high level physical activity (69.3% vs 45.3%) Table 1.

**Table 1:** Characteristics of study participants at enrollment into the CoLTART study, by sex

Characteristic		All [N=1024]	Female [N=669]	Males [N=355]
		n (%)	n (%)	n (%)
Age in years	18-34	111 (10.8)	86 (12.9)	25 (7.0)
	35-49	613 (59.9)	401 (59.9)	212 (59.7)
	50+	300 (29.3)	182 (27.2)	118 (33.2)
Level of education^1^	Incomplete primary/none	459 (45.0)	294 (44.0)	165 (46.7)
	Complete primary	156 (15.3)	104 (15.6)	51 (14.7)
	Secondary +	406 (39.8)	270 (40.4)	136 (38.5)
Occupation^1^	Peasant Farmer	322 (31.6)	205 (30.7)	117 (33.1)
	Gainful employment	210 (20.6)	132 (19.8)	78 (22.1)
	Self-employed / business	408 (40.0)	264 (39.6)	144 (40.4)
	Unemployed	80 ( 7.8)	66 ( 9.9 )	14 ( 4.0 )
SES Score (tertiles)^1^	Low	416 (42.1)	307 (47.8)	109 (31.6)
	Middle	378 (38.3)	240 (47.8)	138 (40.0)
	High	193 (19.6)	95 (14.8)	98 (28.4)
Alcohol consumption^1^	Never	379 (37.5)	277 (41.8)	102 (29.1)
	Ever >1 month ago	366 (36.2)	252 (38.1)	114 (32.6)
	Within < 1 month	267 (26.4)	133 (20.1)	134 (38.3)
Animal proteins consumption (days/week)	0	269 (26.6)	204 (30.8)	65 (18.5)
	1- 2	462 (45.6)	292 (44.1)	170 (48.4)
	3+	282 (27.8)	166 (25.1)	116 (33.0)
Tobacco consumption^2^	Never	843 (82.6)	625 (93.7)	218 (61.8)
	Ex-smoker	97 ( 9.5)	17 ( 2.5 )	80 (22.7)
	Current	80 ( 7.8)	25 ( 3.7 )	55 (15.6)
Physical activity level^2^	Low	473 (46.4)	365 (54.7)	108 (30.7)
	Moderate - high	546 (53.6)	302 (45.3)	244 (69.3)
Total duration on ART (Years)	0-<5	243 (23.7)	150 (22.4)	93 (26.2)
	5-<9	124 (12.1)	74 (11.1)	50 (14.1)
	9+	657 (64.2)	445 (66.5)	212 (59.7)
ART regimen	Non PI based ART	788 (77.0)	519 (77.6)	269 (75.8)
	PI based ART	236 (23.0)	150 (22.4)	86 (24.2)
Cholesterol (>5.2 mol/L)	No	708 (69.8)	442 (66.1)	266 (75.8)
	Yes	306 (30.2)	221 (33.3)	85 (24.2)
HDL (<1 mmol/L)^3^	No	633 (62.4)	378 (57.0)	255 (72.6)
	Yes	381 (37.6)	285 (43.0)	96 (27.4)
LDL (>3.4 mmol/L)^3^	No	775 (76.4)	479 (72.2)	296 (84.3)
	Yes	239 (23.6)	184 (27.8)	55 (15.7)
Triglycerides (>1.69 mmol/L)^3^	No	798 (78.8)	539 (81.3)	259 (74.0)
	Yes	215 (21.2)	124 (18.7)	91 (26.0)
Glucose (>6.0 mmol/L)	No	979 (96.5)	640 (96.5)	339 (96.3)
	Yes	36 ( 3.5)	23 (3.5 )	13 ( 3.7 )
Atherogenic risk (AIP) ^4^ (log_10_(triglycerides/HDL)	No	696 (68.7)	482 (72.7)	214 (61.1)
	Yes	317 (31.3)	181 (27.3)	136 (38.9)
Body Mass Index (kg/m^2^)^5^	<18.5	103 (10.3)	54 ( 8.3 )	49 (14.1)
	18.5–24.9	637 (63.6)	374 (57.2)	263 (75.6)
	>25	282 (26.1)	226 (34.6)	36 (10.3)
Waist/hip ratio (WHR) ^5^ >0.95(men)/>85(women)	Normal	478 (47.4)	207 (31.5)	271 (77.4)
	Abnormal	530 (52.6)	451 (68.5)	79 ( 2.6 )
Hypertension^5^	No	782 (77.4)	505 (76.6)	277 (78.9)
	Yes	228 (22.6)	154 (23.4)	74 (21.1)
Baseline Viral loads^6^ (copies/ml)	< 1000	813 (82.1)	535 (82.8)	278 (80.8)
	1000 – 10,000	53 (5.35)	39 ( 6.0 )	14 ( 4.1 )
	>10,000	124 (12.5)	72 (11.2)	52 (15.1)
Baseline CD4 cell counts^6^ (cells/ml)	< 350	300 (31.5)	163 (24.5)	137 (40.5)
	351 – 500	294 (30.8)	191 (31.0)	103 (30.5)
	> 500	360 (37.7)	262 (42.5)	98 (29.0)

Data was missing on; SES^1^-(27 women and 10 men), occupation^1^ (2 women and 1 man), Level of education^1^(1 woman and 3 men), consumption of Alcohol^1^ and animal protein^1^ (7 women and 5 men). Data was missing for; Tobacco consumption^2^ (2 women and 2 men) and Physical activity^2^ (2 women and 3 men). Data was Missing on; Serum Tc^3^, HDL^3^ LDL^3^, TG^3^ Glucose (6 women and 4 men). Calculation of AIP^4^ excluded 6 women and 3 men with missing data. There was missing data on; HT^5^ (10 Women and 4 men) BMI^5^ (15 women and 7 men), WHR^5^ and WC (11 women and 5 men), Baseline VL^6^ (23 women and 11 men) and Baseline CD4 cell count^6^ ( 53 women, 7 men)

**Prevalence of cardiometabolic risk factors**: Overall, 52.6% had abdominal obesity (high WHR), 26.1% were obese or overweight (BMI ≥25 kg/m2), 30.2% had high Tc, 37.6% had low HDL, 23.6% had high LDL, 21.2% had high TG and 3.5% had hyperglycemia. The prevalence of hypertension was 22.6%, high AIP was 31.6% and 16.6% of the participants had FHS > 10%. There were gender differences in the prevalence of cardiometabolic risk factors; more women than men had; high Tc (33.3% vs 24.2%), low HDL (43.0% vs 27.4%), high LDL (27.8% vs 15.7%), high BMI (34.6% vs 10.3%), abdominal obesity (68.5% vs 22.6%) and abnormal WC (58.1% vs 4.6%). More men than women had: high TG (26.0% vs 18.7%), FHS >10% (30.5% vs 9.2%) and abnormal AIP (38.9% vs 27.3%) Table 1.

**Figure 1:**
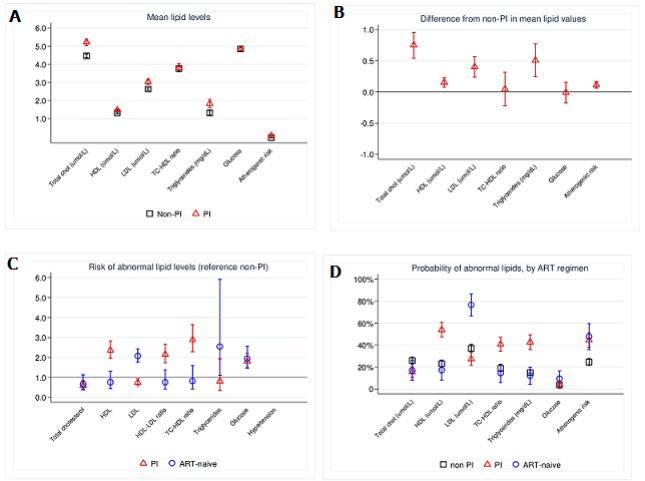
Effect of ART on Cardiometabolic risk factors

**Factors affecting cardiometabolic risk factors among adults on long-term ART**: The PI regimen was significantly associated with: higher mean Tc, HDL, LDL, TG and AIP (P< 0.001) Figure 1 (A, B). Men had significantly lower mean HDL, higher mean TG, Tc:HDL ratios and AIP (P<0.005). Increasing age was significantly associated with higher mean Tc, HDL, LDL (P<0.001) and FBG (P=0.033). Participants who reported consuming animal proteins on at least 3 days in a week had a higher mean Tc (P=0.016), LDL (P<0.004), FBG (P=0.037), FHS (P=0.02), Tc:HDL ratio (P=0.029) and AIP (P=0.022). Duration on ART was associated with increase in mean HDL (P=0.01). Participants in the (P=0.014) and lower Tc: HDL ratio (P=0.005) Table 2. Participants on PI ART had lower SPB (P=0.060) and DBP (P<0.001). Urban site participants had significantly higher SBP (P=0.050) and WC (P<0.001). Men had had significantly higher mean SBP (P=0.004), higher mean WHR, lower mean WC and higher mean FHS (all P<0.001) and lower BMI (P=0.002). We found that increasing age was significantly associated with higher means of SBP, DBP, WHR, WC and FHS (all P<0.001). Increasing intensity of physical activity was significantly associated with lower; SBP, DBP, WC, and FHS (P<0.05). The FHS was significantly higher among current tobacco consumers (P<0.001) but lower among current alcohol consumers (P=0.018) Table 3. We observed lower HDL, higher AIP and FHS, with increasing SES (P<0.05). We observed lower mean HDL, higher mean Tc, LDL, Tc: HDL ratio and AIP with higher HIV viral loads (P<0.001). Increasing CD4 cell counts were associated with higher; HDL (P=0.009) and AIP (P<0.001) Table 2.

**Table 2:** adjusted mean differences in bio-chemical risk factors for cardiovascular disease among adults on long-term ART

Risk Factor		Tc (mmol/L)	HDL (mmol/L)	LDL (mmol/L)	Triglycerides(mmol/L)	Glucose(mmol/L)	Tc: HDL ratio	AIP
		aMD (95% CI)	aMD (95% CI)	aMD (95% CI)	aMD (95% CI)	aMD (95% CI)	aMD (95% CI)	aMD (95% CI)
**ART type**	**P-value**	<0.001	<0.001	<0.001	<0.001	0.902	0.731	<0.001
non-PI		---	---	---	---	---	---	---
PI		0.75 (0.54-0.96)	0.15 (0.07-0.23)	0.40 (0.24-0.57)	0.51 (0.24-0.77)	-0.01 (-0.18-0.16)	0.05 (-0.22-0.32)	0.11 (0.06-0.16)
								
**Duration on ART**	**P-value**	0.550	0.01	0.935	0.909	0.078	0.261	0.423
0-<5 Years		---	---	---	---	---	---	---
5-<9 Years		0.05 (-0.24-0.33)	0.07 (-0.04-0.18)	-0.03 (-0.26-0.20)	0.05 (-0.31-0.42)	-0.00 (-0.23-0.22)	-0.17 (-0.54-0.20)	-0.04 (-0.11-0.03)
9+ Years		0.19 (-0.160.54)	0.20 (0.07-0.33)	0.01 (-0.26-0.29)	0.09 (-0.35-0.54)	0.28 (0.01-0.56)	-0.37 (-0.82-0.08)	-0.05 (-0.14-0.04)
**Study site**	**P-value**	0.301	<0.001	0.014	0.576	0.671	0.005	0.306
Entebbe (peri-rban)		---	---	---	---	---	---	---
Kyamulibwa (Rural**)**		-0.17 (-0.50-0.16)	0.20 (0.07-0.32)	-0.33 (-0.59-0.06)	-0.12 (-0.54-0.30)	-0.06 (-0.32-0.21)	-0.61 (-1.04--0.18)	-0.04 (-0.13-0.04)
**Sex**	**P-value**	0.165	<0.001	0.147	0.005	0,406	<0.001	<0.001
Female		--	--	-	-	--	--	--
Males		-0.14 (-0.34-0.06)	-0.18 (-0.26--0.11)	-0.12 (-0.27-0.04)	0.36 (0.11-0.61)	0.07 (-0.09-0.22)	0.47 (0.22-0.73	0.12 (0.07-0.17)
**Age (years)**	**P-value**	<0.001	<0.001	<0.001	0.691	0.033	0.149	0.456
18-34		---	---	---	---	---	---	---
35-49		0.60 (0.32-0.87)	0.09 (-0.02-0.20)	0.47 (0.25-0.69)	0.15 (-0.20-0.50)	0.21 (-0.01-0.43)	0.33 (-0.03-0.69)	0.04 (-0.03-0.11)
50+		0.96 (0.66-1.26)	0.20 (0.09-0.32)	0.68 (0.44-0.92)	0.15 (-0.23-0.53)	0.31 (0.08-0.55)	0.37 (-0.02-0.75)	0.03 (-0.05-0.10)
**Consumes tobacco**	**P-value**	0.075	0.045	<0.001	0.156	0.128	0.039	0.121
Never		---	---	---	---	---	---	---
Ex-smoker		-0.25 (-0.54-0.05)	0.03 (-0.08-0.15)	-0.38 (-0.62--0.15)	0.32 (-0.06-0.69)	0.01 (-0.22-0.25)	-0.32 (-0.70-0.07)	0.01 (-0.07-0.08)
Current smoker		-0.30 (-0.63-0.02)	0.16 (0.03-0.28)	-0.43 (-0.69--0.17)	-0.14 (-0.56-0.28)	-0.26 (-0.52-0.00)	-0.48 (-0.90--0.05)	-0.08 (-0.17--0.00)
**Consumes alcohol**	**P-value**	0.002	0.002	<0.001	0.202	0.769	0.003	0.155
Never		---	---	---	---	---	---	---
Ever>1month ago		0.30 (0.11-0.49)	0.04 (-0.03-0.11)	0.20 (0.04-0.35)	0.12 (-0.13-0.37)	-0.01 (-0.17-0.14)	0.13 (-0.12-0.38)	-0.00 (-0.05-0.05)
within<1month		-0.01 (-0.23-0.20)	0.11 (0.03-0.20)	-0.10 (-0.27-0.07)	-0.12 (-0.39-0.15)	0.05 (-0.13-0.22)	-0.34 (-0.62--0.06)	-0.05 (-0.10-0.01)
**Animal proteins consumption (days/week)**	**P-value**	0.016	0.341	0.004	0.844	0.037	0.029	0.022
	0	---	---	---	---	---	---	---
	1 or 2	0.02 (-0.18-0.23)	0.06 (-0.02-0.13)	0.00 (-0.16-0.16)	-0.02 (-0.28-0.24)	-0.10 (-0.26-0.06)	-0.08 (-0.35-0.18)	-0.04 (-0.09-0.01)
	3+	0.29 (0.05-0.53)	0.03 (-0.06-0.12)	0.26 (0.07-0.45)	0.05 (-0.25-0.36)	0.10 (-0.09-0.30)	0.27 (-0.05-0.58)	0.02 (-0.04-0.08)
**SES Score (tertiles)**	**P-value**	0.552	0.005	0.415	0.412	0.511	0.111	0.032
Low		---	---	---	---	---	---	---
Medium		0.09 (-0.28-0.09)	-0.10 (-0.17—0.03)	0.04 (-0.19-0.11)	0.17 (-0.08-0.42)	0.08 (-0.08-0.23)	0.20 (-0.04-0.45)	0.05 (0.01-0.10)
High		-0.01 (-0.24-0.22)	-0.11 (-0.20--0.03)	0.08 (-0.10-0.27)	0.06 (-0.25-0.37	0.10 (-0.10-0.29)	0.29 (-0.02-0.59)	0.06 (0.01-0.12)
**CD4counts(cells/ml**	**P-value**	0.782	0.009	0.697	0.055	0.389	0.077	0.001
<350		----	---	----	---	----	----	---
350 – 500		0.05 (-0.16-0.26)	0.02 (-0.06-0.10)	0.06 (-0.11-0.23)	-0.02 (-0.31-0.27)	0.03 (-0.15-0.21)	-0.10 (-0.38-0.18)	0.01 (-0.07-0.04)
>500		0.07 (-0.14-0.29)	0.09 (0.17--0.01	0.07 (-0.10-0.24)	0.28 (-0.01-0.57)	0.12 (-0.06-0.30)	0.20 (-0.08-0.48)	0.07 (0.02-0.13)
**VL (copies ml)**	**P-value**	<0.001	<0.001	<0.001	0.584	0.435	0.001	<0.001
<1000		---	---	---	----	----	---	----
1000 – 9999		0.14 (-0.50-0.23)	-0.22 (-0.36--0.08)	0.03 (-0.26-0.32)	0.01 (-0.48-0.51)	-0.08 (-0.39-0.22	0.46 (-0.03-0.94)	0.09 (0.00-0.18)
10,000+		0.81 (-1.09-0.54)	-0.45 (-0.56--0.35)	0.45 (0.67--0.23)	0.20 (-0.18-0.57)	-0.14 (-0.38-0.09)	0.93 (0.56-1.30)	0.26 (0.19-0.33)

P-value from the likelihood ratio test, aMD - Adjusted mean differences from the reference values (---) , VL – Viral loads, PI-protease inhibitor, SES score-social economic status score, HDL-high density lipoprotein, LDL-low density lipoprotein, Tc-total cholesterol, AIP –Mean Atherogenic Index for Plasma (log10(Triglycerides/HDL).

**Table 3:** Adjusted mean differences (aMD) in bio-physical risk factors for cardiovascular disease among adults on long-term ART

Risk Factor	Systolic BP (mmHg)		Diastolic BP (mmHg)	Waist/hips ratio	WC (cm)	BMI (kg/m^2^)	FHS
	aMD (95% CI)		aMD (95% CI)	aMD (95% CI)	aMD (95% CI)	aMD (95% CI)	aMD (95% CI)
**ART regimen at enrolment**	**p-value**						
	**0.062**	<0.001	0.067	0.267	0.084	0.463	
Non- PI		---	---	---	---	---	---
PI		-3.031 (-6.30-0.2)	-3.771 (-6.3--2.3)	0.010 (-0.001-0.02)	-0.9 (-2.5-0.7)	-1.9 (-4.1-0.3)	-0.3 (-1.2-0.5)
**Study site**	**P-value**	0.050	0.092	0.239	<0.001	0.066	0.125
Entebbe(peri-urban)		---	---	---	---	---	---
Kyamulibwa (rural)		-4.77 (-9.6--0.07)	-2.54 (-5.5--0.45)	-0.010 (-0.027-0.007)	-5.7 (-8.2--3.1)	-3.2 (-6.7-0.2)	-1.0 (-2.3-0.3)
**Sex**	**P-value**	0.004	0.849	<0.001	<0.001	0.002	<0.001
Females	----	---	---	---	---	---	---
Males	----	4.4 (1.4-7.4)	0.2 (-1.7-2.1)	0.034 (0.024-0.044)	-3.4 (-4.9--1.9)	-3.3 (-5.4--1.2)	3.2 (2.3-4.0)
**Age (years)**	**P-value**	<0.001	<0.001	<0.001	<0.001	0.377	<0.001
18-34	----	---	---	---	---	---	---
35-49	----	5.9 (1.7-10.1)	4.3 (1.6-6.9)	0.011 (-0.003-0.025)	3.0 (0.8-5.1)	1.7 (-1.2-4.6)	1.7 (0.5-2.8)
50+	----	15.4 (10.8-20.0)	7.3 (4.5-10.1)	0.028 (0.012-0.043)	4.7 (2.4-7.0)	0.8 (-2.4-3.9)	8.1 (6.9-9.4)
**Animal proteins consumption (days/week)**	**P-value**	0.468	0.336	0.158	0.045	0.161	0.019
	**0**	---	---	---	---	---	---
	**1 or 2**	-0.2 (-3.4-2.9)	-1.1(-3.0-0.8)	-0.009 (-0.019-0.001)	0.7 (-0.9-2.3)	0.7 (-1.4-2.8)	-0.4 (-1.2-0.5)
	**3+**	1.6 (-2.0-5.3)	0.1 (-2.2-2.4)	-0.002 (-0.014-0.010)	2.2 (0.4-4.1)	2.3 (-0.2-4.9)	0.8 (-0.2-1.8)
**Physical activity intensity**	**p-value**	0.014	<0.001	0.859	0.018	0.073	0.010
Low	----	---	---	---	---	---	---
Moderate-High		-3.7 (-6.6--0.7)	-3.9 (-5.7--2.1)	-0.001 (-0.011-0.009)	-1.8 (-3.3--0.3)	-1.8 (-3.9-0.2)	-1.0 (-1.8 - -0.2)
**SES score (tertiles)**	**P-value**	0.483	0.444	0.553	0.134	0.902	0.011
Low	----	---	---	---	---	---	---
Medium	----	-1.25 (-4.20-1.69)	-0.95 (-2.76-0.88)	-0.01(-0.02-0.01)	1.12 (-0.34-2.59)	-0.01 (-0.18-0.16)	0.3 (-0.5 – 1.0)
High		0.80 (-2.85-4.44)	0.29 (-1.96-2.54)	-0.01 (-0.02-0.01)	1.66 (-0.15-3.46)	0.29 (-1.96-2.54)	1.4 (0.5 – 2.3)
**Tobacco consumption**	**P-value**	0.31	0.16	0.85	0.31	0.23	<0.001
Never	----	---	---	---	---	---	---
Ex-smoker	----	0.2 (-4.4-4.7)	0.4 (-2.4-3.2)	0.002 (-0.01-0.02)	-0.6 (-2.9-1.6)	2.6 (-0.5-5.7)	4.3 (3.1-5.5)
Current smoker	----	-3.8 (-8.8-1.2)	-2.8 (-5.9-0.3)	-0.004 (-0.02-0.01)	-1.9 (-4.4-0.6)	1.2 (-2.3-4.6)	3.1 (1.7-4.4)
**Alcohol consumption**	**P-value^+^**	0.56	0.31	0.35	0.20	0.72	0.018
Never	----	---	---	---	---	---	---
Ever>1month ago	----	0.7 (-2.3-3.6)	0.4 (-1.4-2.3)	-0.01 (-0.020-0.003)	-1.1 (-2.5-0.4)	-0.0 (-2.1-2.0)	0.5 (-0.3-1.3)
within<1month		1.8 (-1.5-5.1)	1.5 (-0.5-3.6)	-0.01 (-0.020-0.005)	0.3 (-1.4-1.9)	0.8 (-1.4-3.1)	-0.7 (-1.6-0.2)
**CD4 counts (cells/ml)**	**P-value**	0.18	0.04	0.23	0.02	0.74	0.31
<350	----	----	---	----	----	---	---
350 – 500	----	2.29 (-1.08-5.66)	1.29 (-0.78-3.37)	0.005 (-0.006-0.016)	0.56 (-1.117-2.230)	0.194 (-2.20-2.59)	0.3 (-0.6 – 1.2)
>500		3.09 (-0.28-6.46)	2.68 (0.60-4.76)	0.010 (-0.001-0.021)	2.22 (0.544-3.891	0.88 (-1.52-3.27)	0.7 (-0.2 – 1.5)
**VL (copies /ml)**	**P-value**	0.011	0.073	0.187	0.15	0.81	0.728
<1000	----	-----	----	--	--	--	---
000 – 9999	----	-5.50 (-11.34-0.34)	-1.70 (-5.32-1.92)	0.01 (-0.01-0.03)	2.576 (-0.30-5.45)	-0.39 (-4.92-4.15)	0.6 (-0.9 – 2.1)
10,000+	----	-5.37 (-9.57--1.18)	-2.90 (-5.45--0.26)	0.01 (-0.003-0.02)	1.015 (-1.05 -3.08)	-1.058 (-4.32-2.20)	0.2 (-0.9 – 1.3)

P-value from the likelihood ratio test, aMD -Average mean differences from the reference values ( --- ), PI-protease inhibitor, SES score-social economic status score, HDL-high density lipoprotein, LDL-low density lipoprotein, Tc-total cholesterol, VL-Viral loads, BMI-Body mass index, BP –Blood pressure, WC – Waist circumference and FHS- 10 year Framingham’s risk score

**Effect of PI-containing ART regimen on cardiometabolic risk adjusted for confounders**: Compared to non PI regimens, PI regimen was independently associated with twofold risks of: high Tc (Adjusted risk ratio (aRR): 2.04, 95% CI: 1.67-2.50, P<0.001), high LDL (aRR: 1.70, 95% CI: 1.33-2.16, P<0.001), high TG (aRR: 2.47, 95% CI: 1.90-3.21, P< 0.001) and high AIP (aRR: 1.57, 95% CI: 1.27-1.93, P<0.001). PI regimen had; a 36% less risk of low HDL (aRR: 0.74, 95% CI: 0.59-0.93, P=0.007), a 49% lower risk of hypertension (aRR: 0.51, 95% CI 0.35-0.75, P<0.001), a 16% higher risk of abdominal obesity (aRR: 1.16, 95% CI: 1.02-1.33, P=0.034) and a 41% lower risk of having a FHS >10% (aRR: 0.59, 95% CI: 0.41-0.84, P=0.002) Table 4, Figure 1 (C and D).

**Table 4:** risk of abnormal values of blood pressure and other CVD risk factors

CVD risk factor	1No. abnormal/Total (%)	Risk of abnormal values of cardio metabolic risk factors	Adjusted risk ratio
	Non-PI, n=788 and PI, n=236		P-Value^+^
			(95% CI)
**Total cholesterol ≥5.2 umol/L**	-	-	P<0.001
Non-PI regimen	185/783 (23.6%)	0.25	1
PI regimen	121/231 (52.4%)	0.50	2.04 (1.67-2.5)
**Abnormal HDL**	-	-	P=0.007
Non-PI regimen	313/783 (40.0%)	0.39	1
PI regimen	68/231 (29.4%)	0.29	0.74 (0.59 -0.93 )
**LDL ≥3.4 mmol/L**	-	-	P<0.001
Non-PI regimen	146/783 (18.6%)	0.20	1
PI regimen	93/231(40.3%)	0.34	1.70 (1.33 -2.16 )
**Total cholesterol/ HDL ratio ( >5.1)**	-	-	P=0.938
Non-PI regimen	103/783 (13.1 %)	0.14	1
PI regimen	42/231 (18.2%)	0.14	0.99 (0.68 -1.43 )
**Triglycerides ≥ 1.69 mmol/L**	-	-	P<0.001
Non-PI regimen	116/782 (14.8%)	0.15	1
PI regimen	999/231 (42.9%)	0.38	2.47 (1.90 -3.21 )
**Glucose (>6 mmol/L)**	-	-	P=0.394
Non-PI regimen	20/783 (2.5 %)	0.03	1
PI regimen	5/232 (2.2 %)	0.02	0.63 (0.21 -1.92 )
**Hypertension (SBP ≥140/DBP ≥90) mmHg**	-	-	P<0.001
Non-PI regimen	195/775 (25.2%)	0.25	1
PI regimen	33/235 (14.0%)	0.13	0.51 (0.35 -0.75)
**AIP (log_10_(Triglycerides/HDL) ≥0.1**	-	-	P<0.001
Non-PI regimen	214/782 (27.4%)	0.27	1
PI regimen	103/231 (44.6%)	0.42	1.57 (1.27-1.93
**Abnormal BMI (>25Kg/m^2^)**	-	-	P=0.970
Non-PI regimen	193/770 (25.1%	0.25	1
PI regimen	69/232 (29.7%)	0.25	1.00 (0.78-1.30)
**Abnormal waist circumference [>=94cm(men)/>=80cm(women)]**	-	-	P=0.831
Non-PI regimen	303/773 (39.2%)	0.39	1
PI regimen	95/235 (40.4%)	0.38	0.98 (0.82-1.17)
**Abnormal Waist/hips ratio [>0.95(men)/>85(women)]**	-	-	P=0.034
Non-PI regimen	396/773 (51.2%)	0.49	1
PI regimen	134/235(57.0%)	0.57	1.16 (1.02-1.33)
**Framingham score (>10%)**			P=0.002
Non-PI regimen	138/770 (17.9%)	0.20	1
PI regimen	28/230 (12.2%)	0.12	0.59 (0.41-0.84)

1 A few participants with missing outcome variables. CVD-cardiovascular Disease, PI-protease inhibitor, HDL-high density lipoprotein, LDL-low density lipoprotein, AIP-Atherogenic index for plasma, BMI-Body mass index, P-Value^+^ from the likelihood ratio test Abnormal HDL - HDL<1 mmol/L (Men),<1.3 mmol/L (Females)

## Discussion

In our Ugandan cohort of people on long-term ART, more than a half of participants had abdominal obesity, about one third had high BMI, high Tc, low HDL, high AIP, and almost a quarter had high TG, FHS > 10% and hypertension. Few participants had hyperglycemia. Age, gender, physical activity, high viral load and PI-based ART regimen significantly affected cardiometabolic risk factors. The prevalence of hypertension compares with that reported in a South African study with similar median age [21], but higher than that reported from an ART cohort of younger adults in Rakai-Uganda (8.0%), Mbarara-Uganda (5.2%) and Kenya (8.8%) albeit lower than what was reported from Urban Uganda (27%)(24-26). Although the differences might be attributed to variation in population characteristics and settings, the prevalence exceeded that from the background general population cohort (GPC)-13% [10]. The risk of hypertension was higher among individuals on non PI-based ART. Other factors associated with elevated blood pressure included older age, low physical activity, urbanicity, male gender and lower viral loads. Consistent with other studies, we found a low prevalence of hyperglycemia (3.4%) [24–26], which was not affected by ART regimen [27] but significantly affected by increasing age [28]. The prevalence of abdominal obesity in our study is comparable to the 30% observed in a Southwestern Ugandan ART cohort [26] but was lower than that reported in the background GPC population-56% [10]. High prevalence of obesity among women than men has also been previously established by studies in SSA (24-26) and Europe [29]. The observed risk of obesity from PI-based ART corroborates results from a US based observational study [30] and is possibly attributed to ART associated metabolic aberrations and fat mal-distribution. Studies from developed countries attested to a high risk of abnormal non HDL cholesterol among individuals on PI ART [31]. We observed a higher prevalence of non HDL dyslipidemia but a lower prevalence of low HDL among patients on ART than that reported in the background population [10]. PI's might affect cholesterol metabolism in the liver and in adipocytes [32].The observed association between duration on ART and high mean HDL levels suggests a possible protective effect of long-term ART on aspects of cardiometabolic risk although no literature is available to support this. More men than women had a FHS >10%, which is comparable to findings from a study in central Uganda [33]. The male predominance could be driven by age since men tended to be older, and had higher SBP than women as observed in a US cohort [34].

The higher risk of a FHS >10% among participants on non PI compared to PI regimen could be driven by a high prevalence of hypertension among participants taking non PI ART. The observed non PI ART associated risk of hypertension compares with results from open label ART trials in 7 sub-Saharan African countries [35]. Unlike PI linked vascular complications [36], non PI ART has no known significant associated pathology other than fat misdistribution and dyslipidemia [37]. As ART improves survival, the observed effect of age on Tc:HDL ratio, Tc, LDL and hypertension highlights the potential risk of CVD in a population aging on ART. Age induced dyslipidemia is thought to be due to increased inflammation due to high levels of Tumor necrosis alpha (TNF- α) and interleukin-6 (IL-6) which interfere with lipid metabolism. Our men had higher mean TG consistent with studies in South Africa and the US [24, 38]. We chose AIP as cardiovascular risk stratification to assess the influence of atherogenic dyslipidemia (TG and LDL) in predicting coronary atherosclerosis and cardiovascular risk in both hypertensive and normotensive individuals [22]. Higher mean AIP in men has also been reported among HIV patients in Nigeria [39], probably due to the elevated TG among men. Consumption of animal protein was associated with elevation of both TG and AIP. The PI regimen was associated with higher risk of abnormal AIP probably due to PI associated dyslipidemia. Participants in the urban study site had significantly higher mean LDL and Tc: HDL SBP, DBP, WC and BMI than those from the rural study site. This is in line with findings in the general population linking increasing urbanicity to higher prevalence of CVD risk factors [40], underscoring the importance of the ongoing rural-urban transition [41]. The association between HIV viraemia and dyslipidemia had been demonstrated previously. Viraemia is thought to mediate dyslipidemia through promoting peroxidation which is responsible for alteration of cholesterol metabolism [42]. Our analysis was based on a relatively large cohort of participants who had been on ART for almost a decade which enabled assessment of long-term effects of ART on cardiometabolic risk. We measured fasting blood lipid and glucose levels which minimized misclassification. The wide age range and a relatively large number of individual level exposures minimized confounding. We used both AIP and FHS as a comprehensive way of stratifying cardiovascular risk prediction, although the value of these scales has not been assessed among HIV infected people in Africa. Prior screening for CVD and education against CVD risk factors may have influenced self-reported data such as physical activity alcohol, salt and tobacco consumption. We defined variables using Western reference cut offs, however our findings corroborate findings from other studies. In calculating the FHS, left ventricular hypertrophy was not radiologically assessed.

## Conclusion

Cardiometabolic risk is an increasingly important co-morbidity among people on ART in SSA countries including Uganda. Both the PI and non PI ART are associated with increased cardiometabolic risk. The protective effect of PI on hypertension and HDL dyslipidemia deserves further investigation. Increased HIV viral load testing in SSA, will detect more PLWH on first line ART needing second line PI based ART. Integration of routine CVD risk assessment and preventive treatments into HIV care programs is recommended to prevent cardiovascular related mortality and morbidity. Emphasis on protective social-behavioral and dietary modifications is also important especially in urban settings. The findings also underscore the need to investigate ART associated cardiovascular risk in children. Our findings will inform policy on target interventions in HIV care to reduce cardiovascular associated morbidity and mortality among HIV-positive people on ART in sub-Saharan African settings

### What is known about this topic

- HIV and ART increase cardiometabolic risk;
- The PI regimen is known to increase cardiometabolic risk;
- Duration on ART is associated with cardiometabolic risk.

### What this study adds

- In SSA Antiretroviral Therapy irrespective of type of regimen is associated with cardiometabolic risk;
- Duration on ART does not independently increase cardiometabolic risk in African settings;
- With the advent of test and treat, routine cardiovascular risk assessment should be integrated into HIV care programs.

## Competing interests

The authors declare no competing interest.

## References

[1] UNAIDS Global Statistics Facts sheet.

[2] Liu C, Ostrow D, Detels R, Hu Z, Johnson L, Kingsley L (2006). Impacts of HIV infection and HAART use on quality of life. Quality of Life Research.

[3] Goosby E, Dybul M, Fauci A A, Fu J, Walsh T, Needle R (2012). The United States President's Emergency Plan for AIDS Relief: a story of partnerships and smart investments to turn the tide of the global AIDS pandemic. JAIDS Journal of Acquired Immune Deficiency Syndromes.

[4] Triant VA, Lee H, Hadigan C, Grinspoon SK (2007). Increased acute myocardial infarction rates and cardiovascular risk factors among patients with human immunodeficiency virus disease. The Journal of Clinical Endocrinology & Metabolism..

[5] Mutimura E, Crowther N J, Stewart A, Todd Cade W (2008). The human immunodeficiency virus and the cardiometabolic syndrome in the developing world: an African perspective. Journal of the cardiometabolic syndrome.

[6] WHO GENEVA Global Health estimates summary tables: Projection of deaths by cause, age and sex, by World Bank Income Group and WHO region 2013.

[7] World Health Organization (2009). Global health risks: mortality and burden of disease attributable to selected major risks.

[8] Dolan SE, Hadigan C, Killilea KM, Sullivan MP, Hemphill L, Lees R S (2005). Increased cardiovascular disease risk indices in HIV-infected women. JAIDS Journal of Acquired Immune Deficiency Syndromes.

[9] Lundgren JD, Battegay M, Behrens G, De Wit S, Guaraldi G, Katlama C (2008). European AIDS Clinical Society (EACS) guidelines on the prevention and management of metabolic diseases in HIV. HIV medicine.

[10] Asiki G, Murphy GA, Baisley K, Nsubuga RN, Karabarinde A, Newton R (2015). Prevalence of dyslipidaemia and associated risk factors in a rural population in South-Western Uganda: a community based survey. PLoS One.

[11] Buchacz K, Weidle PJ, Moore D, Were W, Mermin J, Downing R (2008). Changes in lipid profile over 24 months among adults on first-line highly active antiretroviral therapy in the home-based AIDS care program in rural Uganda. JAIDS Journal of Acquired Immune Deficiency Syndromes.

[12] Commission UA (2014). HIV and AIDS Uganda Country Progress Report 2013.

[13] Uganda MoH Addendum to the National Antiretroviral Treatment Guidelines for Uganda.

[14] Gibb DM, Kizito H, Russell EC, Chidziva E, Zalwango E, Nalumenya R (2012). Pregnancy and infant outcomes among HIV-infected women taking long-term ART with and without tenofovir in the DART trial. PLoS Med..

[15] Kazooba P, Kasamba I, Baisley K, Mayanja B N, Maher D (2012). Access to, and uptake of, antiretroviral therapy in a developing country with high HIV prevalence: a population-based cohort study in rural Uganda, 2004-2008. Tropical medicine & international health.

[16] Organization WH (2015). STEPwise approach to noncommunicable disease risk factor surveillance (STEPS).

[17] Murphy GA, Asiki G, Ekoru K, Nsubuga RN, Nakiyingi-Miiro J, Young EH (2013). Sociodemographic distribution of non-communicable disease risk factors in rural Uganda: a cross-sectional study. International journal of epidemiology.

[18] Alberti G, Zimmet P, Shaw J, Grundy SM (2006). The IDF consensus worldwide definition of the metabolic syndrome. Brussels: International Diabetes Federation.

[19] Expert Panel on Detection E (2001). Executive summary of the Third Report of the National Cholesterol Education Program (NCEP) expert panel on detection, evaluation, and treatment of high blood cholesterol in adults (Adult Treatment Panel III). Jama..

[20] James PA, Oparil S, Carter BL, Cushman WC, Dennison-Himmelfarb C, Handler J (2014). 2014 evidence-based guideline for the management of high blood pressure in adults: report from the panel members appointed to the Eighth Joint National Committee (JNC 8). Jama..

[21] Mashinya F, Alberts M, Colebunders R (2015). Assessment of cardiovascular risk factors in people with HIV infection treated with ART in rural South Africa: a cross sectional study. AIDS research and therapy.

[22] Dobiássová M, Frohlich J (2001). The plasma parameter log (TG/HDL-C) as an atherogenic index: correlation with lipoprotein particle size and esterification rate inapoB-lipoprotein-depleted plasma (FER HDL). Clinical biochemistry.

[23] Payne R (2010). The University of Edinburgh. Cardiovascular Risk Calculator.

[24] Clark SJ, Gómez-Olivé FX, Houle B, Thorogood M, Klipstein-Grobusch K, Angotti N (2015). Cardiometabolic disease risk and HIV status in rural South Africa: establishing a baseline. BMC public health.

[25] Sander LD, Newell K, Ssebbowa P, Serwadda D, Quinn TC, Gray RH (2015). Hypertension, cardiovascular risk factors and antihypertensive medication utilisation among HIV-infected individuals in Rakai, Uganda. Tropical Medicine & International Health.

[26] Muyanja D, Muzoora C, Muyingo A, Muyindike W, Siedner M J (2016). High prevalence of metabolic syndrome and cardiovascular disease risk among people With HIV on stable ART in Southwestern Uganda. AIDS patient care and STDs..

[27] Dillon DG, Gurdasani D, Riha J, Ekoru K, Asiki G, Mayanja BN (2013). Association of HIV and ART with cardiometabolic traits in sub-Saharan Africa: a systematic review and meta-analysis. International journal of epidemiology.

[28] Omech B, Sempa J, Castelnuovo B, Opio K, Otim M, Mayanja-Kizza H (2012). Prevalence of HIV-associated metabolic abnormalities among patients taking first-line antiretroviral therapy in Uganda. ISRN AIDS..

[29] McCormick CL, Francis AM, Iliffe K, Webb H, Douch CJ, Pakianathan M (2013). Increasing Obesity in Treated Female HIV Patients from Sub-Saharan Africa: Potential Causes and Possible Targets for Intervention. Frontiers in immunology.

[30] Lombo B, Alkhalil I, Golden M P, Fotjadhi I, Ravi S, Virata M (2015). Prevalence of metabolic syndrome in patients with HIV in the era of highly active antiretroviral therapy. Conn Med..

[31] Badiou S, De Boever CM, Dupuy A, Baillat V, Cristol J, Reynes J (2003). Decrease in LDL size in HIV-positive adults before and after lopinavir/ritonavir-containing regimen: an index of atherogenicity. Atherosclerosis.

[32] Feeney ER (2011). Mallon PW. HIV and HAART-associated dyslipidemia. Open Cardiovascular Medicine Journal.

[33] Mateen F J, Kanters S, Kalyesubula R, Mukasa B, Kawuma E, Kengne AP (2013). Hypertension prevalence and Framingham risk score stratification in a large HIV-positive cohort in Uganda. Journal of hypertension.

[34] Marma AK, Berry JD, Ning H, Persell SD, Lloyd-Jones DM (2010). Distribution of 10-year and lifetime predicted risks for cardiovascular disease in US adults. Circ Cardiovasc Qual Outcomes.

[35] Shaffer D, Hughes MD, Sawe F, Bao Y, Moses A, Hogg E (1999). Cardiovascular Disease Risk Factors in HIV-Infected Women Following Initiation of Lopinavir/ritonavir-and Nevirapine-based Antiretroviral Therapy in Sub-Saharan Africa: A5208 (“OCTANE”). Journal of acquired immune deficiency syndromes.

[36] Vittecoq D, Escaut L, Monsuez J (1998). Vascular complications associated with use of HIV protease inhibitors. The Lancet.

[37] Yone EWP, Betyoumin A F, Kengne A P, Folefack FJK, Ngogang J (2011). First-line antiretroviral therapy and dyslipidemia in people living with HIV-1 in Cameroon: a cross-sectional study. AIDS Research and Therapy.

[38] Cook CB, Erdman DM, Ryan GJ, Greenlund KJ, Giles WH, Gallina DL (2000). The pattern of dyslipidemia among urban African-Americans with type 2 diabetes. Diabetes care.

[39] Onyedum CC, Young EE, Iroezindu MO, Chukwuka CJ, Nwagha UI (2014). Atherogenic index of plasma in highly active antiretroviral therapy-naïve patients with human immunodeficiency virus infection in Southeast Nigeria. Indian journal of endocrinology and metabolism.

[40] Riha J, Karabarinde A, Ssenyomo G, Allender S, Asiki G, Kamali A (2014). Urbanicity and lifestyle risk factors for cardiometabolic diseases in rural Uganda: a cross-sectional study. PLoS Med..

[41] Cohen B (2006). Urbanization in developing countries: Current trends, future projections, and key challenges for sustainability. Technology in Society.

[42] Constans J, Pellegrin J, Peuchant E, Thomas M, Dumon M, Sergeant C (1992). Membrane fatty acids and blood antioxidants in 77 patients with HIV infection. La Revue de medecine interne/fondee par la Societe nationale francaise de medecine interne.

